# Employment of the Triple Helix concept for development of regenerative medicine applications based on human pluripotent stem cells

**DOI:** 10.1186/2001-1326-3-9

**Published:** 2014-05-09

**Authors:** Peter Sartipy, Petter Björquist

**Affiliations:** 1 Cellectis AB, Arvid Wallgrens Backe 20, 413 46 Göteborg, Sweden; 2 Systems Biology Research Center, School of Bioscience, University of Skövde, 541 28 Skövde, Sweden; 3 Current address: NovaHep AB, Arvid Wallgrens Backe 20, 413 46 Göteborg, Sweden

**Keywords:** Human pluripotent stem cells, Clinical applications, Regenerative medicine

## Abstract

Using human pluripotent stem cells as a source to generate differentiated progenies for regenerative medicine applications has attracted substantial interest during recent years. Having the capability to produce large quantities of human cells that can replace damaged tissue due to disease or injury opens novel avenues for relieving symptoms and also potentially offers cures for many severe human diseases. Although tremendous advancements have been made, there is still much research and development left before human pluripotent stem cell derived products can be made available for cell therapy applications. In order to speed up the development processes, we argue strongly in favor of cross-disciplinary collaborative efforts which have many advantages, especially in a relatively new field such as regenerative medicine based on human pluripotent stem cells. In this review, we aim to illustrate how some of the hurdles for bringing human pluripotent stem cell derivatives from bench-to-bed can be effectively addressed through the establishment of collaborative programs involving academic institutions, biotech industries, and pharmaceutical companies. By taking advantage of the strengths from each organization, innovation and productivity can be maximized from a resource perspective and thus, the chances of successfully bringing novel regenerative medicine treatment options to patients increase.

## Introduction

Human pluripotent stem cells (hPSC) can be isolated from in vitro fertilized eggs or they can be generated from somatic cells through the process referred to as re-programming [1,2]. Established hPSC lines can be propagated indefinitely and the cells can differentiate into virtually any specialized cell type of the adult. The ability to harness these unique properties of hPSC forms the foundation for the tremendous expectations on the applications of these cells, and their derivatives, for treating human diseases which are characterized by tissue degeneration and cell loss. Over the last decade our knowledge about how to maintain and expand undifferentiated hPSC has improved substantially. In parallel, the understanding of hPSC differentiation on a molecular- and mechanistic level has increased dramatically, resulting in the establishment of efficient differentiation protocols for several human cell types [3]. When combining this know-how, it is easy to appreciate that the expectations on hPSC in the area of regenerative medicine are high, since the possibility to create large amounts of human cell types seems to be just around the corner. These expectations are in many ways valid; however, there are still substantial hurdles that need to be overcome before regenerative medicine applications based on hPSC could reach the market.

Human pluripotent stem cells have started to find their way into the clinical setting and we have witnessed rapid progress in this field during recent years. The first FDA-approved clinical trial initiated by Geron (http://www.geron.com) in 2009 using their cell therapy product GRNOPC1, a population of living cells containing oligodendrocyte progenitor cells derived from human embryonic stem cells (hESC), targeted spinal cord injury. Although this study was halted for financial reasons in 2011, the patients that already had received the cellular transplants will be monitored for a period of 15 years according to the original study protocol using public state funding. Two other studies initiated by Advanced Cell Technology (http://www.advancedcell.com) have received FDA-approval for transplantation of their cell therapy product, MA09-hRPE, to patients with Advanced Dry Age Related Macular Degeneration or Stargardt's Macular Dystrophy. MA09-hRPE is a population of hESC-derived retinal pigment epithelium cells and the hope is to be able to slow or halt the process of blindness associated with these degenerative diseases. Initial safety data have been published and appear promising [4]. In parallel, the Japanese authorities have given their approval to the first clinical study using human induced pluripotent stem cell (hiPSC)-derived cells. This investigation, led by researchers at the RIKEN Center for Developmental Biology (http://www.riken.jp), Kobe, Japan, is targeting patients suffering from exudative (wet-type) Advanced Macular Degeneration. The study will take advantage of a clinically compliant protocol involving the establishment of autologous hiPSCs from each of the study subjects and a differentiation method to generate retinal pigment epithelium cells for subsequent transplantation back to the patients. Although these recent developments are exciting and could represent the beginning of a new era in regenerative medicine, concerns have been raised, and it has been argued that some safety issues require additional investigations in order to better assess the risks involved when transplanting hPSC-derived cells to humans [5]. Indeed, for the whole field of hPSC-based cell therapy it is a lot at stake, and failure of the initial clinical trials due to safety reasons will have substantial negative impact on any future developments in this space. One can expect that these pioneering clinical studies will be monitored closely by the regulatory agencies, scientists, investors/financers, as well as patient groups and the general public.

**Figure 1:**
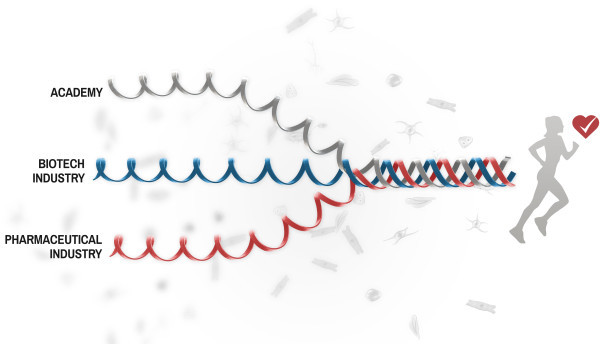
**The Triple Helix concept.** Cartoon illustrating the Triple Helix model depicting how academia, biotech industry (SME), and pharmaceutical industry may work close together in order to synergize development of novel regenerative medicine applications for the improvement of human health.

For everyone involved in stem cell based regenerative medicine projects it is clear that bringing new hPSC-based therapies from bench-to-bed is an extremely complex and costly process. Challenges ranges from scientific/technical- to clinical- and regulatory- as well as financial/business development related hurdles. One way to address complex challenges is to establish cross-disciplinary collaborations and set up teams which are equipped to address each of these challenges in an efficient way. This requires access to specialist competencies from individuals belonging to academic research centers, small and medium size biotech, as well as large pharma and regulatory agencies. The Triple Helix concept typically refers to the hybridization between university, industry, and government to generate knowledge and innovation [6]. In our model described here (Figure 1), we include university, small- and medium size enterprises (SME) (i.e., biotech industry), and large pharmaceutical companies in the Triple Helix, and we will illustrate how benefits and strengths can be captured from each organization in order to drive the development of therapeutic applications of hPSC.

## Review

### Setting up cross-disciplinary collaborations for regenerative medicine programs

Forming cross-disciplinary collaborations may at a first glance appear challenging. However, by appropriately matching complementary skills and interests, the process can be straight forward. Although each partner may have separate short term goals, the foundation in the program must be focused on the final long term goal which is to cure patients and improving quality-of-life for individuals who are suffering from, as-of-yet, untreatable degenerative diseases. The main advantage of bringing together expertise from academic institutes, SME, and large pharma into one collaborative program is that one can capture the benefits and strengths from each organization in a timely fashion. In the following section, we aim to illustrate this by exemplifying some of the challenges of bringing hPSC-based therapies from bench to bed and propose how these issues can be addressed through creative interdisciplinary collaborations.

The criteria for identifying key partners in industry and academia should be comprised of several important aspects. The project team should include access to scientific and technical expertise in a number of areas including developmental biology, stem cell differentiation, cell production, and process development including quality assurance and quality control. From the medical side it is important to bring in experts and key-opinion-leaders in the disease area that is being targeted in order to have in-depth understanding of the disease mechanisms and pathology, and also knowledge of the currently available treatments, if there are any. Other important competencies to include are from clinical development and the regulatory side. Based on the recent advent of this field, the experience from hPSC-based therapeutic applications is today limited both from the clinical- as well as from the regulatory aspects. This makes it important to follow the developments closely and having the opportunity to adapt quickly to any alterations in the clinical and regulatory landscape that may arise. It is expected that adjustments of the program need to be done as the developments progress. Thus, it is critical that the project team is highly flexible and can adapt to new conditions, both based on internal advancements but also based on possible changes in the external environment and regulatory landscape. For example, it was recently highlighted that most of the hESC lines listed on the NIH Stem Cell Registry do not comply with US Food and Drug Administration’s requirements for commercializable starting material for medicinal products [7]. Notably, this is due to the restricted use of NIH funds for the derivation and use of hESC lines which is not compatible with the FDA regulations for biologically generated therapeutic products for human use. In Europe, the situation is similar, and funds from the European Commission cannot be used for the derivation of new hESC lines, which will be needed in order to bring hESC-based therapies to the market. Thus, research programs relying on such federal- and international grants are currently facing substantial regulatory hurdles and these issues must be resolved. Investments in product development and commercialization using non-FDA approved hESC-lines seem unlikely from the industry side since these efforts may need to be repeated using a starting material that fulfils the FDA regulations.

One advantage of adopting the modified Triple Helix concept consisting of the academic institute, SME, and the pharmaceutical industry is that the risk of internal competition between the organizations in the project team can be minimized. In a simplistic view, one could consider the role of the academic researchers to be focused mainly on the basic science aspects of the project, such as furthering the fundamental understanding of the molecular processes involved in cell differentiation to specific lineages and deciphering the mechanisms critical for cell survival and functional integration post-transplantation. It should be noted that many academic institutions have become increasingly interested in supporting intellectual property protection by their researchers. This may appear as an obstacle for industrial implementation since it may involve negotiating terms and conditions for technology licensing also with the academic institute. However, investments from the industry is preferable done in areas with freedom to operate and patent protection, thus having a unique and protected technology would rather increase the interest from industry partners. While the academic partners would focus on the basic science, the SME, on the other hand, would undertake the industrial implementation and transfer the knowledge generated at the academic institute and perform tasks such as standardization of processes and quality control. Another typical role for the SME partner would be to head the refinement of enabling assets such as culturing media, up-scaling technologies for undifferentiated and differentiated cells, as well as derivation of new hPSC lines of GMP grade. Finally, animal experimentation, pre-clinical toxicity testing, and clinical development entails large resources and an organization to meet regulatory requirements, and is today probably best led by large pharma. It may appear that the different partners have discrete roles, which could certainly be the case in some situations, but the key aspect here is still close collaboration. In order for the project to move forward efficiently it is imperative that all partners have detailed insights into each other’s activities. Regular joint project meetings are a simple tool to stimulate discussions and interactions between the project teams and increase transparency between the partners. In addition, fostering and supporting an environment that encourages interactions on all levels, from management to bench researchers, clearly have positive effects for generating a creative and constructive atmosphere which is a crucial factor for a successful project progression. Especially in the process of transferring knowledge and technologies from one organization to another, part of the success depends on the opportunity of having key individuals physically following the implementation phase on a practical level. Indeed, tech transfer is often problematic in life science and biotech due to the delicate procedures involved when handling biological material. In particular, culturing and differentiating hPSCs are complex processes and transferring methodologies across laboratories are challenging [8]. However, by taking advantage of the cross-disciplinary approach described here we believe that the strength of the Triple Helix model is highly beneficial for the development of clinical applications based on hPSC.

### Addressing hPSC-challenges with cross-disciplinary teams

Although major advancements have been achieved over the last decade related to the culture and directed differentiation of hPSC, we are still at the early stages of understanding on a detailed level how these processes are governed. For instance, our knowledge about the phenomenon of pluripotency remains limited. The clues we have at hand, thus far, involve an insight into the transcriptional machinery that regulate and to some extent control the pluripotent state [9]. Transcription factors, such as Oct4, Sox2, Nanog, Klf-4, and c-Myc, have all been observed to be critical for maintaining the pluripotent state of hESC, and also been shown to be instrumental for the reprogramming of somatic cells into iPSC [2]. A much more thorough understanding of the pluripotent state will be important to improve and better define culturing systems for large scale production of homogenous undifferentiated hPSC and to make these cells ready for directed differentiation into specialized cells. It should be noted that for the indications currently targeted in the ongoing and planned clinical trials using hPSC-derived retinal pigment epithelium cells, the number of cells that are transplanted to the patients are relatively low (about 50,000 cells) [4], but the cell dosage expected for other indications such as diabetes and heart disease are in the 10 billion range [10]. Taking these cell numbers into the calculation, it is clear that currently used culturing systems will be far from sufficient and bioreactors or other types of large volume suspension culturing systems must be developed to meet the needs from patients. Such developments require the collaborative effort between basic stem cell biologist, engineers, and experts in bioprocessing and would fit well into the Triple Helix model. For example, an SME developing culture vessels or bioreactors would instantly get access to specialized knowledge in stem cell biology through collaborations with academic teams and on the other side they would have a bench-marking partner in the large pharma which could provide critical feed-back on new developments as they progress. Notably, many times the pharmaceutical industry is entering regenerative medicine from being experts in a certain disease area but without detailed knowledge about hPSC technologies per se. Here, the SMEs and academic teams may play an important role as experts in the field bringing key aspects of this new technology into the larger company’s organization and procedures. An example of successful transfer of technologies from academy to industry is the clinical applications of hESC-derived oligodendrocyte progenitors which originated from University of California at Irvine and later led to the use of these cells for implantation in human spinal cord injuries in the clinical study by Geron mentioned above [11].

The derivation and culture of clinically compliant hPSC has been described [12] but limitations in scale of production and high costs remain significant challenges. In addition to the technical issues involved, for ethical reasons, it is important to establish the donor consent documentation appropriately and utilize forms that specifically states that the cells will be used for cell therapy purposes. We have witnessed many examples of hPSC lines which have been derived using donor consent forms that only cover “in vitro research use” of the cells and consequently prevent the lines to be used in a clinical and commercial setting. Thus, we believe that it will be required in the future to derive new hPSC lines, of clinical grade, using appropriate donor consent. Although the hESC-lines used in the clinical trials described above have received post-derivation approval for clinical use, we believe that for broader use, future hPSC lines should be derived under strict GMP conditions from the start. That is, these cell lines should be derived strictly both feeder- and xeno-free in order to be compatible with future requirements from the regulatory agencies world-wide. Such cell lines are still not available, but this work is in progress in our own laboratories as well as in others. Critical bottlenecks to be addressed include the development of standardized feeder- and xeno free conditions for establishment of hPSC lines encompassing identification of qualified reagents, which is a non-trivial task. Nevertheless, recent reports have shown that the establishment of iPSC lines can be done without genetic manipulation and this process may greatly simplify regulatory compliance in the future [13]. Furthermore, differentiation protocols that may fulfill the criteria set for GMP production have also been described for various cell types including retinal pigment epithelium cells [4], vascular cells [14], and neurons [15]. However, substantial additional basic research is needed to develop a cell therapy product based on hPSC, and in this setting the collaboration between academia and industry is important. Taking advantage of the deep knowledge in developmental biology and stem cell differentiation available at the Universities and combining this with early input from industry will pave the way for efficient establishment of industry-ready protocols for cell production. Today, directed hPSC differentiation is typically achieved by sequentially exposing the cells in a delicate time- and dose dependent manner to growth factors and signaling molecules that enriches the final cultures for a specific cell type, which may or may not require further purification. In most cases, the methodologies developed at the research bench necessitate substantial modifications to reach the criteria needed for industrial implementation. For example, for GMP production, it is a formal requirement to have traceable and qualified reagents as well as defined culture medium. In addition, the cost-of-goods is an important aspect which is often overlooked at the early stages of protocol development in small scale. Before going into large scale process development it is highly advantageous to have defined differentiation protocols in which expensive protein growth factors have been replaced by less expensive alternatives (e.g., small molecules). There are several examples in which this has been successfully accomplished both for establishment of hPSC lines and for directed differentiation efforts [16]. A thorough understanding of the basic mechanisms involved in cell type specific differentiation is needed to efficiently make this transition to reduce the cost-of-goods. From a practical perspective, handling and differentiating hPSC have traditionally encompassed substantial manual labor and this is not compatible with large scale production where automation is a key factor. Simplifying the practical moments and reducing the physical manipulations of the cell cultures are also issues that are important to deal with at an early stage of protocol development and cross-disciplinary collaborations are well equipped to address these challenges.

Besides the challenges in setting up a production platform that can deliver an hPSC-based therapeutic product at an acceptable cost and scale, it is important to define the optimal type of hPSC-derivative to finally transplant. Grafting undifferentiated hPSC is associated with a substantial risk of tumor formation [17] and depending on the disease it may be appropriate to derive a committed progenitor population or a more fully differentiated mature phenotype. Taking the example of diabetes, it has been reported in pre-clinical animal models that by xeno-grafting pancreatic endoderm derived from hESC, it was possible to cure animals with experimentally induced diabetes [18]. This strategy is now further explored and developed towards a clinical application by Viacyte using their PEC-01™ cells (http://www.viacyte.com). An alternative approach, currently explored in our ongoing program together with Novo Nordisk (http://www.novonordisk.com) and Lund University (Sweden), is to develop fully functional glucose-responsive β-cells and in the end to transplant these cells to patients using an encapsulation technique. In principal, there are pros and cons with both strategies. The progenitor strategy encompasses the possibility of in situ cell expansion, having the benefit of requiring smaller cell doses and one may speculate that progenitors are able to survive and functionally integrate post-transplantation more efficiently than more mature cells. On the other hand, the fully differentiated cell therapy product may represent a “safer” product since it is a post-mitotic cell population (i.e., low risk of tumor formation) which can be more precisely dosed. Encapsulation of islet cell grafts has been successfully reported both in animal models and human subjects [19,20]. In the specific situation of cell therapy for diabetes, it is not necessary to achieve functional integration with the host tissue since the cells are aimed to serve as glucose sensor and insulin producers, and their contact only via with the blood circulation should be sufficient. For diabetes treatment using cell therapy, the consensus today is that the cells should be encapsulated in order be protected from the immune system of the host, but at the same time allowing nutrients and hormones to cross the membrane. The exact site of transplantation is still under consideration but the liver or in the subcutaneous space has been proposed to be suitable sites. However, most experimental cell transplantations are delivered directly to the site of injury or in an adjacent region by injecting a small volume of a cell suspension using fine needles or glass capillaries. Alternatives include a systemic approach via intravenous infusions. Besides encapsulation, other options to avoid a devastating immune reaction would be to generate banks of matching cell lines (so-called “haplobanks”) or to engineer the genome of cells to cause less response from the host by for example knocking out immune-reaction causing antigens [21]. The use of mesenchymal stromal cells as immunomodulatory agents also represents an alternative to suppress immune reactions caused by implanted stem cell derivatives [22,23]. In each specific disease indication it is critical to assess which strategy is most appropriate and many factors need to be taken into consideration. Thus, the cross-disciplinary approach clearly has benefits in these situations where aspects related to defining cell product maturation stage, administration and delivery strategy need to be evaluated in the context of engraftment capacity, homing, survival, activation of the immune system, and functional integration of the cells at the site of injury.

When getting closer to the clinical situation, issues related to quality control and testing of the final cell therapy product are important. It is likely that development of quality standards and characterization methods for each hPSC-based therapeutic product and intended clinical application will be needed. Adding to the complexity of defining and characterizing the cell therapy product is the possibility for using mixed populations of cells to achieve the therapeutic effect. A pure population of only one cell type, which could be easier to define, may be less potent and effective. The potency testing is critical for the development of hPSC-based cell therapy products and is needed before progression to phase III clinical trials. However, a strict potency assay, ideally a product’s in vivo mechanism of action, may not be possible since the product may encompass complex multiple functions, some of which are not well defined. Most likely, novel potency assays needs to be developed and this requires innovation and basic research in combination with standardization and industrial quality systems and the pharma industry has a key role in these processes. Relevant pre-clinical testing in vivo requires well deigned and suitable animal models. Common for all hPSC-derived cell therapy products is that one needs to consider effects that relate to xeno-transplantation when testing the human cells in animal models. How this parameter will affect the interpretation of the results must be addressed on a case-to-case basis and is at this stage difficult to predict. Nevertheless, establishment of general strategies and guidelines on how to assess potency and safety of hPSC-derived cellular products is critical, and close interactions with the regulatory agencies are very important in order to safely translate experimental cell therapy to the clinic. The choice of trial design and regulatory issues may also cause difficulties and lead to clinical trial failures, further underscoring the importance of the close interactions with the regulatory agencies already at an early stage.

Development of hPSC-based cell therapies are expected to be long and expensive and clinical failure can be due to other factors besides efficacy and safety. We have already witnessed termination of clinical programs due to financial reasons and business strategies (http://www.geron.com). A collaborative project between academy, SME, and large pharma can take advantage of different funding mechanisms and there are opportunities to tap into various external sources of support. Funding for basic research carried out in the academic setting is available, on competitive grounds, from national research councils and institutes (e.g., National Institutes of Health in the US and Medical Research Council in the UK). In addition, many other organizations, active nationally or globally, are also supporting research towards hPSC-based cell therapy (e.g., Juvenile Diabetes Research Foundation, http://www.jdrf.org). In the private sector, SMEs have the possibility to attract venture capital to fund efforts in regenerative medicine. In addition and perhaps most importantly, the pharmaceutical companies have many times the possibility to directly support the project using their internal resources. Besides individually applying for grants or securing other type of funding, the partners in the Triple Helix model can also join together and obtain consortium grants or support based on collaborative networks from international initiatives such as the Framework Programs funded by the European Union. In the US, the NIH also has specific funding programs that promote risky research collaborations between industry and academia, such as the Small Business Innovation Research (SBIR) and Small Business Technology Transfer (STTR) Programs. In addition, the Technology Strategy Board in the UK recently established Cell Therapy Catapult (http://www.ct.catapult.org.uk), a not-for-profit organization, with the aim to help businesses take innovative ideas in the area of cell therapy through to commercialization. This is one example of how governmental initiatives can help taking products into the clinic and de-risking them for further investment. Taken together, having partners aligned with the Triple Helix model, which cover both non-profit and for-profit organizations, is of great advantage when developing hPSC-based cell therapy applications since this will allow the flexibility to fund the research and development using a variety of sources decreasing the financial risk for the project for the individual partners.

## Conclusions

Most fruitful and successful collaborations are characterized by the “win-win situation” where all partners have clear benefits of working together and they share a mutual trust and respect and an openness to share information and data among participating partners. In the challenging process of developing novel hPSC-based therapeutic applications it is imperative to establish collaborations and engage expertise from various disciplines and organizations. The Triple Helix model appears well suited for addressing some of the challenges, and the benefits for each organization can be identified. For example, through the course of a successful hPSC-based therapy project, research data will be generated that most likely will be possible to publish in high-impact journals which will profile the academic researchers in the field and form the foundation for future grant applications. The SMEs can benefit from the program since they can secure important intellectual property and build their business and technology platform to become the partner-of-choice also in other hPSC-based therapeutic programs. In addition, the SMEs may become the manufacturer of the final cell therapy product as well as of other assets needed, such as cell lines and media of clinical grade, or enabling tools for e.g. encapsulation or administration. Finally, as the provider of the cell therapy, the pharmaceutical company will sustain their business in the health care system and build their capacity to meet the novel era which is anticipated to radically change how diseases are treated today. Together, the partners of the Triple Helix model will change the landscape for future regenerative medicine applications with a speed that would have been impossible for any of the partners operating in isolation.

## Abbreviations

hPSC: Human pluripotent stem cells; hESC: Human embryonic stem cells; hiPSC: Human induced pluripotent stem cell; SME: Small- and medium size enterprises.

## Competing interests

Peter Sartipy and Petter Björquist are employed by Cellectis AB (http://www.cellectis.com).

## Authors’ contributions

PS and PB: conception and design, manuscript writing, final approval of manuscript.
